# Effect of Regulation of Whole-Plant Corn Silage Inoculated with *Lactobacillus buchneri* or *Bacillus licheniformis* Regarding the Dynamics of Bacterial and Fungal Communities on Aerobic Stability

**DOI:** 10.3390/plants13111471

**Published:** 2024-05-26

**Authors:** Hang Yin, Meirong Zhao, Rui Yang, Juanjuan Sun, Zhu Yu, Chunsheng Bai, Yanlin Xue

**Affiliations:** 1 College of Horticulture, Shenyang Agricultural University, Shenyang 110866, China; 2 Institute of Grassland Research, Chinese Academy of Agricultural Sciences, Hohhot 010010, China; 3 College of Grassland Science and Technology, China Agricultural University, Beijing 100193, China; 4 Inner Mongolia Engineering Research Center of Development and Utilization of Microbial Resources in Silage, Inner Mongolia Academy of Agriculture and Animal Husbandry Science, Hohhot 010031, China

**Keywords:** whole-plant corn silage, aerobic stability, *Lactobacillus buchneri*, *Bacillus licheniformis*, bacterial community, fungal community

## Abstract

Enhancing the aerobic stability of whole-plant corn silage is essential for producing high-quality silage. Our research assessed the effect of inoculation with *Lactobacillus buchneri* or *Bacillus licheniformis* and its modulation of the bacterial and fungal microbial community structure in an aerobic stage of whole-plant corn silage. Following treatment with a distilled sterile water control, *Lactobacillus buchneri*, and *Bacillus licheniformis* (2 × 10^5^ cfu/g), whole-plant corn was ensiled for 60 days. Samples were taken on days 0, 3, and 7 of aerobic exposure, and the results showed that inoculation with *Lactobacillus buchneri* or *Bacillus licheniformis* improved the aerobic stability of silage when compared to the effect of the control (*p* < 0.05). Inoculation with *Bacillus licheniformis* attenuated the increase in pH value and the decrease in lactic acid in the aerobic stage (*p* < 0.05), reducing the filamentous fungal counts. On the other hand, inoculation with *Lactobacillus buchneri* or *Bacillus licheniformis* increased the diversity of the fungal communities (*p* < 0.05), complicating the correlation between bacteria or fungi, reducing the relative abundance of *Acetobacter* and *Paenibacillus* in bacterial communities, and inhibiting the tendency of *Monascus* to replace *Issatchenkia* in fungal communities, thus delaying the aerobic spoilage process. Due to the prevention of the development of aerobic spoilage microorganisms, the silage injected with *Lactobacillus buchneri* or *Bacillus licheniformis* exhibited improved aerobic stability.

## 1. Introduction

Silage is now widely used worldwide as a ruminant feed; fermentation under anaerobic conditions allows it to reduce the total loss of nutrients from harvest to storage [1,2]. However, when the stored silage is opened for feeding, aerobic microorganisms proliferate rapidly, and acids and water-soluble carbohydrates are oxidized to carbon dioxide and water [3,4,5,6], leading to increased temperature and silage deterioration. The aerobic deterioration of silage can produce toxic substances that are harmful to both animals and humans [7]; feeding ruminants with aerobically spoiled silage can lead to a reduced nutritional intake [8]. Therefore, slowing down aerobic spoilage is a key issue in silage preparation.

Whole-plant corn silage plays a major role as a feed for dairy cattle around the world as a valuable source of energy and nutrients [9,10]. However, with its high residual soluble carbohydrate content and the lactic acid it produces during fermentation, it provides ideal conditions for yeasts and filamentous fungi to multiply, causing aerobic deterioration when whole-plant corn silage is exposed to oxygen [11]. Compared to lactic acid, acetic acid is more resistant to fungal fermentation [4]. Additionally, inoculants of heterofermentative lactic acid bacteria, such as *Lactobacillus buchneri*, can convert soluble carbohydrates into acetic acid during fermentation and inhibit the activity of filamentous fungi and yeasts, improving the aerobic stability of whole-plant corn silage [12,13]. However, this method is less efficient at preserving nutrients than is non-inoculation [14]. Recently, it was found that *Bacillus subtilis* inoculation can produce antifungal and antibacterial substances in silage that can inhibit fungal growth, thereby enhancing its aerobic stability [15,16], which provides new possibilities for maintaining fermentation quality while improving the aerobic stability. It has been shown that the environment, consumers of products from animals that have been fed with treated silage, and the target species are all thought to be safe around *Bacillus licheniformis* DSM 32457 [17], but its effectiveness in silage has yet to be verified. Research by Afordoanyi et al. [18] has shown that combining corn silage with homogeneous fermenting lactic acid bacteria and *Bacillus licheniformis* strain WJ53A reduces the risk of Clostridium perfringens infection in animal husbandry. Similarly, the study by Zhu et al. [19] showed that inoculation with *Bacillus licheniformis* could improve silage quality. As *B. licheniformis* and *B. subtilis* belong to the same genus *Bacillus*, it was speculated that inoculation with *B. licheniformis* could also improve the aerobic stability of silage. However, these results in regards to whole-plant corn silage need to be verified.

Using next-generation sequencing methods, it has been discovered that microbial inoculants increase the aerobic stability of silage by altering the structure of the microbial community [20]. According to Wang et al. [21], treatment with *Lactobacillus hilgardii* and mixed inoculation with *Lactobacillus plantarum* in sugarcane top silage inhibited the growth of spoilage microbes that are linked to aerobic deterioration, such as *Acetobacter pasteurianus* and *Paenibacillus amylolyticus*. A study by Liu et al. [22] used *Lactobacillus buchneri* to inhibit the growth of *Penicillium* and *Monascus* in *Leymus chinensis* silage, improving aerobic stability, while Yin et al. [23] observed that *B. subtilis* inoculation suppressed the relative abundance of bacteria and fungi such as *Bacillus* and *Kazachastania* during aerobic exposure in whole-plant corn silage. The effect of *B. licheniformis* inoculation on the bacterial community structure of hybrid *Pennisetum* silage was investigated by Zhu et al. [19], who revealed that inoculation improved the silage quality by increasing the relative abundance of *Lactobacillus*. However, less information is available regarding the *L. buchneri* or *B. licheniformis* microbial communities on whole-plant corn silage. Therefore, the objective of this study was to examine the impact of *B. licheniformis* or *L. buchneri* on the aerobic stability, fermentation quality, and bacterial and fungal communities of whole-plant corn silage under aerobic exposure conditions.

## 2. Results

### 2.1. Chemical Composition of Fresh Material and Nutrient Composition of Whole-Plant Corn Silage

Prior to ensiling, the counts of lactic acid bacteria, coliform bacteria, and yeasts were 5.64, 5.90, and 6.39 lg CFU/g, respectively; counts of filamentous fungi were below the detection threshold. The concentrations of DM, CP, aNDF, ADF, and WSC were 372.54 g/kg FW, 79.84, 375.72, 173.84, and 113.70 g/kg DM, respectively (Table 1).

The BL group had a lower ash content than the CK group (*p* < 0.05); there was no significant difference found in the contents of DM, CP, aNDF, ADF, and WSC between the groups (*p* > 0.05; Table 2).

### 2.2. Aerobic Stability

**Figure 1:**
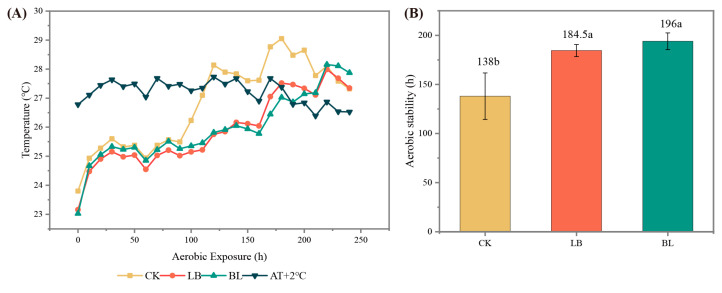
Dynamics of silage temperature (°C) (**A**) and aerobic stability (**B**) during aerobic exposure. Means with different lowercase letters (a,b) indicate significant differences in aerobic stability time (*p* < 0.05). AT + 2 °C represents ambient temperature plus 2 °C. CK represents silage without inoculation; LB represents silage with inoculation of *Lactobacillus buchneri*; BL represents silage with inoculation of *Bacillus licheniformis*.

The LB and BL treatment groups exhibited a higher aerobic stability than did the CK group (Figure 1).

**Figure 2:**
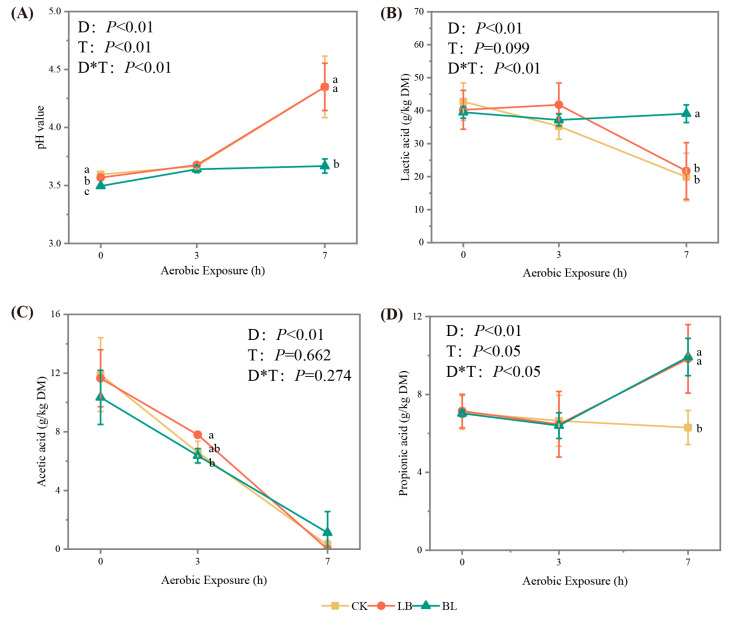
pH (**A**), lactic acid (**B**), acetic acid (**C**), and propanoic acid (**D**) of silage during aerobic exposure. Means with different lowercase letters (a,b) differ significantly (*p* < 0.05). D represents aerobic exposure days; T represents treatments; D*T represents interaction between treatments and aerobic exposure days. CK represents silage without inoculation; LB represents silage with inoculation of *Lactobacillus buchneri*; BL represents silage with inoculation of *Bacillus licheniformis*.

The inoculants had an influence on pH and propionic acid contents (*p* < 0.05); the duration of aerobic exposure had an effect on pH, as well as the lactic acid, acetic acid, and propionic acid contents (*p* < 0.05; Figure 2); the interaction of the two had an effect on pH, as well as the lactic acid and propionic acid contents (*p* < 0.05). When the aerobic exposure duration was extended, the pH values and lactic acid contents of each group progressively increased and decreased, respectively. However, on day 7 of the aerobic exposure, the pH value of the LB and BL groups was lower than that of the CK group (*p* < 0.05), the propionic acid content was higher than that of the CK group (*p* < 0.05), and the lactic acid content of the BL group was higher than that of the CK group (*p* < 0.05). In addition, the acetic acid concentration of the LB group was greater than that of the CK group on day 3 of aerobic exposure (*p* < 0.05).

**Figure 3:**
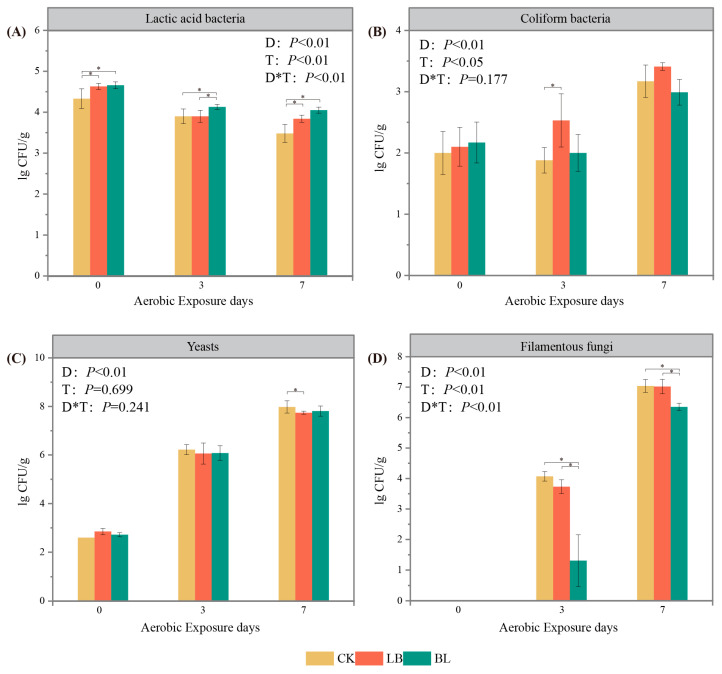
Lactic acid bacteria (**A**), coliform bacteria (**B**), yeasts (**C**), and filamentous fungi (**D**) of silage during aerobic exposure. * significance at *p* < 0.05. D represents aerobic exposure days; T represents treatments; D*T represents interaction between treatments and aerobic exposure days. CK represents silage without inoculation; LB represents silage with inoculation of *Lactobacillus buchneri*; BL represents silage with inoculation of *Bacillus licheniformis*.

The number of lactic acid bacteria, coliform bacteria, and filamentous fungi was influenced by both the aerobic exposure time and the inoculant treatment (*p* < 0.05; Figure 3); yeast was also influenced by aerobic exposure time (*p* < 0.05), while the numbers of lactic acid bacteria and filamentous fungi were influenced by their interactions (*p* < 0.05). In all groups, with the duration of aerobic exposure, the quantity of filamentous fungus, yeasts, and coliform bacteria steadily rose, while the number of lactic acid bacteria gradually declined. On days 0 and 7 of aerobic exposure, there were more lactic acid bacteria in the LB and BL groups than in the CK group (*p* < 0.05), while, on day 7 of aerobic exposure, there were fewer filamentous fungi in the BL group than in the CK group (*p* < 0.05).

### 2.3. Microbial Community

For both bacteria and fungi, all the treatment groups exhibited coverage values higher than 0.99. With an increase in the aerobic exposure period, the bacteria in each group exhibited a drop in the Shannon and Chao indices (*p* < 0.05) and an increase in the Simpson index (*p* < 0.05) (Table 3). On day 7 of aerobic exposure, the Shannon index was lower in the BL group than in the CK and LB groups, the Chao index was lower in the LB and BL groups than in the CK group (*p* < 0.05), and the Simpson index showed no significant difference.

With the prolongation of aerobic exposure, the Shannon and Chao indices of the fungal community groups gradually decreased, while the Simpson index increased (*p* < 0.05; Table 4). After 7 days of aerobic exposure, the BL group showed a higher Shannon index than the CK group, the LB group had a lower Chao index than the CK and BL groups (*p* < 0.05), and the LB and BL groups had a lower Simpson index than the CK group (*p* < 0.05).

**Figure 4:**
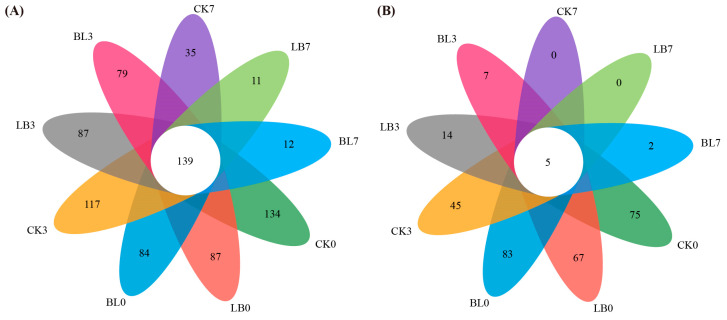
Venn diagram of bacterial (**A**) and fungal (**B**) OTUs in silage during aerobic exposure. CK0, CK3, and CK7 represent silage not inoculated at 0 d, 3 d, and 7 d of the aerobic exposure stage, respectively; LB0, LB3, and LB7 represent silage inoculated with *Lactobacillus buchneri* at 0 d, 3 d, and 7 d of the aerobic exposure stage, respectively; BL0, BL3, and BL7 represent silage inoculated with *Bacillus licheniformis* at 0 d, 3 d, and 7 d of the aerobic exposure stage, respectively.

OTU clustering of the sequences, based on a 97% similarity threshold (Figure 4), showed that the treated bacterial samples shared 139 OTUs overall, whereas CK0 expressed more OTUs during aerobic exposure, with 134 (Figure 4A). All treated fungi samples shared 5 OTUs, while BL0 exhibited more OTUs overall (Figure 4B).

**Figure 5:**
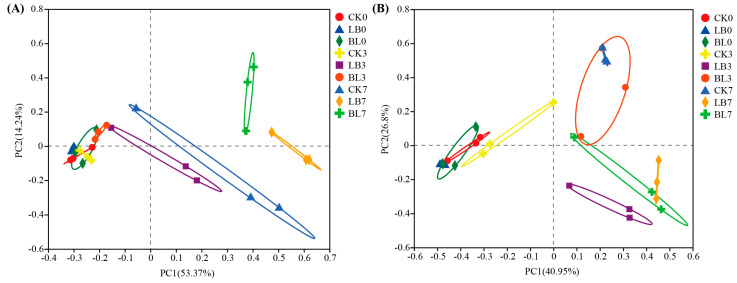
Principal coordinate analysis of bacterial (**A**) and fungal (**B**) communities in silage during aerobic exposure. CK0, CK3, and CK7 represent silage not inoculated at 0 d, 3 d, and 7 d of the aerobic exposure stage, respectively; LB0, LB3, and LB7 represent silage inoculated with *Lactobacillus buchneri* at 0 d, 3 d, and 7 d of the aerobic exposure stage, respectively; BL0, BL3, and BL7 represent silage inoculated with *Bacillus licheniformis* at 0 d, 3 d, and 7 d of the aerobic exposure stage, respectively.

There was a clear separation of the bacterial community on day 7 versus days 0 and 3 of aerobic exposure (Figure 5A). In contrast, for the fungal community, there was a noticeable separation between days 0 and 7 of aerobic exposure (Figure 5B), where CK7 was separated from LB7 and BL7, respectively.

**Figure 6:**
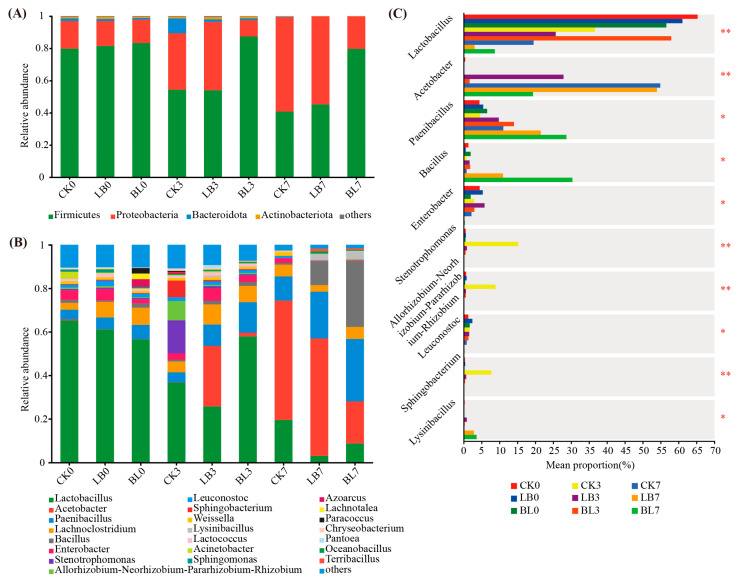
Changes of bacteria at the phylum level (**A**), genus level (**B**), and the differences in genus level (**C**). * significance at *p* < 0.05; ** significance at *p* < 0.01. CK0, CK3, and CK7 represent silage not inoculated at 0 d, 3 d, and 7 d of the aerobic exposure stage, respectively; LB0, LB3, and LB7 represent silage inoculated with *Lactobacillus buchneri* at 0 d, 3 d, and 7 d of the aerobic exposure stage, respectively; BL0, BL3, and BL7 represent silage inoculated with *Bacillus licheniformis* at 0 d, 3 d, and 7 d of the aerobic exposure stage, respectively.

At the phylum level, *Firmicutes* and *Proteobacteria* dominated the bacterial community in the aerobically exposed whole-plant corn silage. The relative abundance of *Proteobacteria* increased over the 7 days of aerobic exposure, although it did so more slowly in the BL group than in the CK group (Figure 6A). As the aerobic exposure duration increased, the relative abundance of *Lactobacillus* decreased gradually (Figure 6B). On day 3 of aerobic exposure, the CK group had a higher relative abundance of *Stenotrophomonas* than the LB and BL groups (*p* < 0.05; Figure 6C), while on day 7, the BL group had a lower relative abundance of *Acetobacter* than the CK and LB groups (*p* < 0.05). However, the relative abundances of *Paenibacillus* and *Bacillus* were higher than those of both the CK and LB groups (*p* < 0.05).

**Figure 7:**
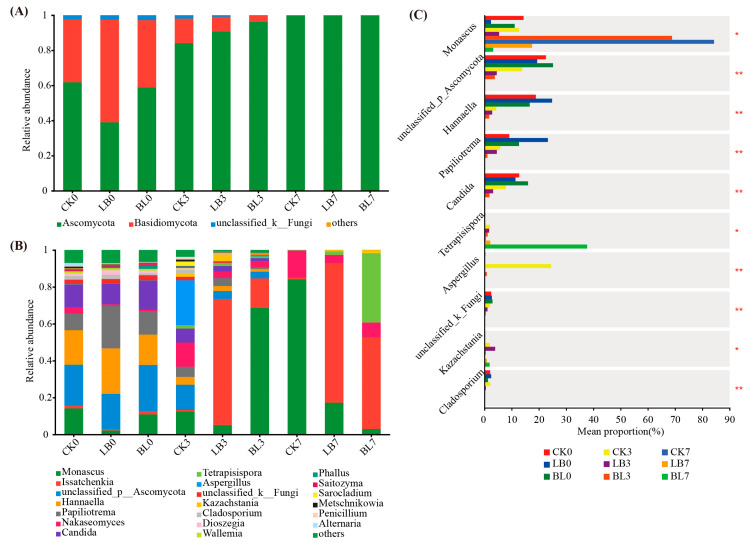
Changes in fungi at the phylum level (**A**), genus level (**B**), and the differences in genus level (**C**). * significance at *p* < 0.05; ** significance at *p* < 0.01. CK0, CK3, and CK7 represent silage not inoculated at 0 d, 3 d, and 7 d of the aerobic exposure stage, respectively; LB0, LB3, and LB7 represent silage inoculated with *Lactobacillus buchneri* at 0 d, 3 d, and 7 d of the aerobic exposure stage, respectively; BL0, BL3, and BL7 represent silage inoculated with *Bacillus licheniformis* at 0 d, 3 d, and 7 d of the aerobic exposure stage, respectively.

*Ascomycota* and *Basidiomycota* mostly regulated whole-plant corn silage during aerobic exposure, with *Ascomycota* increasing as the duration of aerobic exposure increased (Figure 7A). The dominant genera in each group on day 0 of aerobic exposure were *Monascus*, *unclassified_p_Ascomycota*, *Hannaella*, *Papilotrema*, and *Candida* (Figure 7B). *Monascus* and *Issatchenkia* showed a gradual increase in relative abundance during aerobic exposure, with the LB7 and BL7 groups having a lower relative abundance of *Monascus* than the CK7 group (*p* < 0.05; Figure 7C), whereas the BL3 group had a greater relative abundance of *Monascus* than the CK3 and LB3 groups (*p* < 0.05). The relative abundance of *Tetrapisispora* was higher in the BL7 group than in the CK7 and LB7 groups (*p* < 0.05).

**Figure 8:**
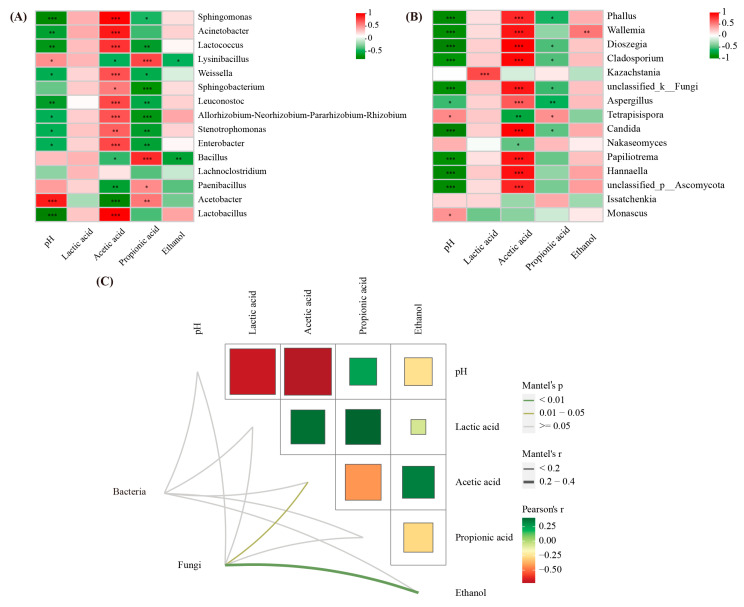
Spearman analysis between silage parameters and bacterial species (**A**), and fungal species (**B**) during aerobic exposure; Mantel tests were used to analyze the correlations of fermentation characteristics and the abundance of bacterial and fungal communities (**C**). * significance at *p* < 0.05; ** significance at *p* < 0.01; *** significance at *p* < 0.001.

The pH value is positively correlated with the abundance of *Acetobacter* (*p* < 0.001), while it is negatively correlated with the abundances of *Lactobacillus*, *Enterobacter*, *Stenotrophomonas*, etc. in the bacterial community (*p* < 0.05; Figure 8A). The acetic acid content of the silage is positively correlated with the abundance of *Lactobacillus*, *Enterobacter*, etc. (*p* < 0.001), but negatively correlated with the abundance of *Acetobacter*, *Paenibacillus*, etc. (*p* < 0.01), while propionic acid demonstrates the opposite phenomenon. In the fungal community, the pH value of the silage is significantly positively correlated with the abundance of *Monascus* and *Tetreapisispora* (*p* < 0.01), whereas it is negatively correlated with the abundance of *Hannaella*, *Candida*, etc. (*p* < 0.001; Figure 8B). The contents of lactic acid are positively correlated with the abundance of *Kazachstania* (*p* < 0.001), while the contents of acetic acid are positively correlated with the abundance of *Hannaella*, *Candida*, etc. (*p* < 0.001). The Mantel test revealed that the primary variables influencing the alteration of the fungal community after the aerobic exposure of whole-plant corn silage were the concentrations of ethanol and acetic acid (*p* < 0.05; Figure 8C).

**Figure 9:**
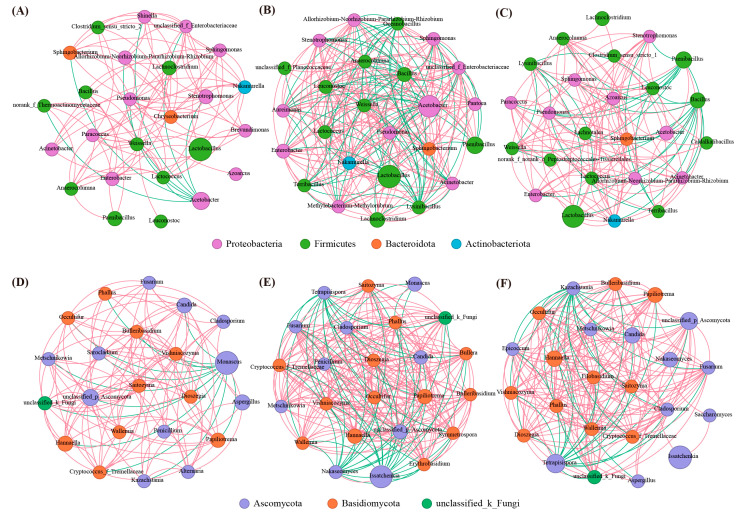
Interaction networks of the silage in bacterial (**A**–**C**) and fungal (**D**–**F**) communities. (**A**,**C**) represent silage not inoculated; (**B**,**E**) represent silage inoculated with *Lactobacillus buchneri*; (**C**,**F**) represent silage inoculated with *Bacillus licheniformis*. Node size and color represent the bacterial phylum and relative abundance, respectively; the edges are colored based on negative (green) and positive (red) correlations.

Regarding the interspecies correlation of bacterial and fungal communities, the numbers of nodes and edges in the LB and BL groups in bacteria and fungi were higher than those of the CK group (Figure 9).

## 3. Discussion

### 3.1. Characteristics of Fresh Material and Nutrient Composition of Silage

The DM content in this experiment is within the recommended range (32–38%) [24], and the contents of DM, CP, aNDF, ADF, and WSC did not significantly differ across the groups, indicating that inoculation with *Lactobacillus buchneri* or *Bacillus licheniformis* does not reduce the nutrient content of whole-plant corn silage.

### 3.2. Aerobic Stability

Compared to those in the CK group, it was found that the aerobic stabilization times of the LB and BL groups were longer, suggesting that LB or BL inoculation might enhance aerobic stability. An essential metric for assessing the fermentation impact of silage is the pH value [25]; LB0 and BL0 had pH values that were lower than those of CK0 (*p* < 0.05), and in many studies, inoculation with LB increases the pH value [26,27]. However, the results of some studies demonstrate that inoculation with LB resulted in a decrease in pH value [28,29,30]. In contrast to CK7, the pH value of the BL7 group was lower (*p* < 0.05), probably due to the fact that, at this time, the lactic acid bacteria number of BL7 was higher than that of the CK group (*p* < 0.05), and the number of filamentous fungi was lower than in the CK group (*p* < 0.05). Aerobic microorganisms use lactic acid as a substrate for their growth after aerobic exposure in silage, causing the pH of the silage to rise [31]; a high pH circumstance is favorable for the growth of filamentous fungi, while the proliferation of filamentous fungi also causes an increase in silage temperature [4]. This explains why the aerobic stability of the BL group was superior to that of the CK group.

According to the study of Ferrero et al. [32], aerobic stability is closely related to yeast counts, and it decreases exponentially with increasing yeast population [33]. Furthermore, inoculation with LB has been shown to inhibit the proliferation of undesirable microorganisms by increasing the acetic acid content, thus improving the aerobic stability of silage [34,35]. In this experiment, the Mantel analysis showed that fungal communities were mainly correlated with acetic acid content, and that the counts of yeast in LB7 were lower than those in CK7 (*p* < 0.05), which might account for the better aerobic stability of the LB group.

The activity of aerobic microorganisms, including filamentous fungus and yeasts, is inhibited by propionic acid [36] and in whole-plant corn silage, it has been shown to improve aerobic stability as an additive in experiments such as those conducted by Chen et al. [5]. In this experiment, BL7 and LB7 exhibited a higher propionic acid content than CK7 (*p* < 0.05). In addition, the counts of yeasts in LB7 and filamentous fungi in BL7 were significantly lower than in the CK7 group (*p* < 0.05), which contributed to the delay in the onset of aerobic decay in the LB and BL groups.

### 3.3. Microbial Community

The coverage values of all treatment groups in regards to bacteria and fungi were greater than 0.99, indicating that sequencing has adequately captured most bacterial and fungal communities. With prolonged exposure to oxygen, the Shannon and Chao indices of the bacterial and fungal communities in each treatment decreased, while the Simpson index increased (*p* < 0.05), indicating a decline in both the diversity and abundance of the bacterial and fungal communities [21]; this result is consistent with what has been found in previous research [20], which implies that aerobic microorganisms that are tolerant to low amounts of acid proliferate in whole-plant corn silage [30] and become relatively simple during aerobic exposure. In comparison with CK7, in the fungal community, the Chao and Shannon indexes of the BL7 group were higher, while the Simpson index was lower (*p* < 0.05), which suggests that inoculation with BL maintains the diversity of fungal communities during aerobic exposure. We speculate that this may explain the improved aerobic stability in the BL group, consistent with our previous results of higher fungal community diversity and better aerobic stability in whole-plant corn silage that is inoculated with LB or BS [23]. In contrast, in the bacterial community, BL7 had lower Chao and Shannon indices compared to CK7, suggesting that inoculation with BL reduced the diversity of this community, which could be attributed to the fact that BL7 has a lower pH [20,37]. BL7 has a higher number of unique OTUs than CK7, which may also explain its improved aerobic stability. Separation of the bacterial and fungal populations in the latter phases of aerobic exposure between the inoculation treatment group (LB or BL) and the non-inoculated group (CK) in principal coordinate analysis suggests that by changing the structure of the bacterial and fungal communities, the two microbial inoculants increase aerobic stability.

Bacterial communities in aerobically exposed whole-plant corn silage were dominated by *Firmicutes* and *Proteobacteria*, with *Proteobacteria* gradually becoming the more dominant bacterial community, which is in line with the results of previous experiments [38]. In a study by Sun et al. [39], at the genus level, the dominance of lactic acid bacteria was gradually replaced by other aerobic microorganisms with increasing aerobic exposure time; this result is similar to that obtained by our study. *Stenotrophomonas* was one of the aerobic bacteria used in corn stover silage in the study by Liu et al. [40], whereby the results of the experiment found clear support for the relative abundance of *Stenotrophomonas* at CK3 than at LB3 and BL3 (*p* < 0.05), which suggested that the bacterial community in the CK group evolved towards aerobic bacteria. We observed an increase in the relative abundance of *Paenibacillus* at 3 d and 7 d of aerobic exposure; a similar pattern of results was obtained in a study by Wang et al. [21]. They determined that in sugarcane top silage, an increase in *Paenibacillus* was observed for a period of time after the onset of aerobic spoilage. It was found that *Acetobacter* can induce the aerobic spoilage of silage, gradually dominating the bacterial community [21]; in this study, the relative abundance of *Acetobacter* on day 7 of aerobic exposure was increased in all groups compared to days 0 and 3, but the relative abundance of BL7 was smaller than that of CK7 (*p* < 0.05). In the correlation analysis, *Acetobacter* was negatively correlated with pH value, which may be the reason for the delay in its aerobic spoilage. *Bacillus* is one of the aerobic bacteria that cause the aerobic spoilage of silage [39] and its relative abundance was higher in BL7 and LB7 than in CK7 (*p* < 0.05). However, it did not result in aerobic spoilage, probably due to the fact that *Bacillus* is involved in the successional process and competitively slows down the relative abundance of *Acetobacter* and *Paenibacillus*.

*Ascomycota* and *Basidiomycota* mostly controlled whole-plant corn silage in the aerobic stage, this result ties in well with those of previous studies by Liu et al. [41]. *Issatchenkia* is an important microbiota that is associated with silage spoilage [42]; in this trial, *Issatchenkia* dominates when exposed to oxygen on days 5 to 7. During the later stages of aerobic exposure in the fungal community tested in the present study, a process of succession from yeasts, represented by *Issatchenkia*, to filamentous fungi, represented by *Monascus*, occurred, which could be slowed down via the inoculation of LB or BS. A period of *Issatchenkia* dominance was not observed in CK, and we speculate that this might be due to the fact that it is possible that in CK, this period occurs between 3 and 7 d, without setting up a sampling site. *Monascus*, which may use a variety of carbohydrates and acids as carbon sources and may generate a wide range of enzymes to break down organic materials [22], is associated with aerobic spoilage, and it is the dominant filamentous fungus genus in aerobically exposed silage [43]. In this experiment, the relative abundance of *Monascus* was higher in CK7 than in LB7 and BL7 (*p* < 0.05), and it is positively correlated with pH value (*p* < 0.05), which may explain the faster aerobic decay in CK. BL3 had a higher relative abundance of *Monascus* than CK3 and LB3 (*p* < 0.05), but is lowered for BL7, which also did not result in decay on day 3 of aerobic exposure, possibly due to the filamentous fungi count of BL3 being 1.31 lg CFU/g, which was significantly lower than that of CK3 and LB3. At this time, the yeast population is overwhelmingly dominant, and the succession of dominant fungi from yeast to filamentous fungi has not yet occurred. In BL7, *Monascus* was replaced by *Tetrapisispora*, which has been shown in several studies to secrete a killer toxin (Kpkt) for application in the winemaking process [44]. Kitamoto et al. [45] screened for killer yeast strains in yeast genera such as *Kluyveromyces* and *Saccharomyces* to inhibit the growth of wild yeasts and thus, to inhibit the aerobic spoilage of silage. Therefore, we speculated that it would produce Kpkt in silage in a similar manner, inhibiting the activity of other undesirable fungi, and thus delaying the onset of aerobic spoilage even more so in BL compared to LB treatments.

The higher the number of nodes and edges, the lower the level of intermediary and categorical variables, and the higher the network complexity [46]. The correlation between species was constructed using the top 25 most abundant genus level bacterial and fungal networks in the aerobic exposure phase, and the results showed that different inoculants alter the correlation in fungal, LB, and BL inoculations, making the correlation more complicated. This is consistent with the observation that both treatments had a lower Simpson’s index than CK in the fungal alpha diversity analysis. Additionally, the study of Liu et al. [42] has confirmed that silage exposed to oxygen still maintains a high microbial complexity, favoring the retardation of aerobic spoilage.

## 4. Materials and Methods

### 4.1. Materials and Silage Preparation

The Baicaoyuan Scientific Research Base of Shenyang Agricultural University in Liaoning, China (41°50′ N, 123°34′ E) provided the whole-plant corn (Liaodan 5802) raw materials for this experiment. When the corn reached the wax maturity stage, it was harvested, leaving a 15 cm stubble height, chopped to 1–2 cm by a grass shredding machine (Donghong No. 1, Donghong Mechanical Equipment Co., Ltd., Zhengzhou, China). *Bacillus licheniformis* groups (BL, 2 × 10^5^ cfu/g), *Lactobacillus buchneri* (LB, 2 × 10^5^ cfu/g), and a distilled sterile water control (CK) were the methods used to treat silage. After melting the inoculant in sterile water and evenly spraying it, the ingredients were completely mixed with additives. Then, about 600 g were placed within vacuum-sealed silage bags (35 cm by 24 cm) and were sealed using a vacuum packaging machine. For the purpose of fermentation, all samples were kept protected from light for 60 days at room temperature (23–28 °C) and underwent three repetitions for every group.

### 4.2. Aerobic Stability

Once the bag was opened, a 180 g sample of each treatment was moved into a Styrofoam block-encased 250 mL beaker. According to the methods of Tao et al. [47], to verify aerobic stability, a 64-well thermograph probe was inserted into the opened silage core. Data were obtained every 10 min. After being exposed to oxygen for 0, 3, and 7 days, the silage was sampled in order to examine the microbial communities, aerobic stability, and quality of fermentation. The aerobic spoilage of silage is measured by a rise in silage temperature of more than 2 °C above ambient temperature [32].

### 4.3. Fermentation Quality and Nutrient Composition Analyses

The 100 g silage samples were acquired after 60 days, and after 48 h of drying at 65 °C, the dry matter (DM) content was determined. Using an Ankom 2000 fiber analyzer (Ankom, Macedon, NY, USA), the neutral detergent fiber content (assayed with a heat stable amylase, or aNDF) and acid detergent fiber (ADF) contents were determined, in accordance with the methodology described by Van Soest et al. [48]. A Kjeldahl technique [49] was used to determine the crude protein (CP) level, and an autoanalyzer (Kjeltec 8400; FOSS Co., Ltd., Hillerød, Denmark) was utilized. Water-soluble carbohydrate (WSC) content was measured using colorimetry, following a reaction with the anthrone reagent [50].

The 20 g silage samples were combined with 180 mL of sterile water and refrigerated for 18 h at 4 °C. Using a pH meter with a glass electrode (PB-10, Sartorius Group, Göttingen, Germany), the pH of the extract was determined [11]. Using a 210 nm UV detector (Waters2414, Waters Corporation, Milford, MA, USA) and a chromatographic column (Sepax Technologies, Inc., Newark, DE, USA), high-performance liquid chromatography (Waters1525, Waters Corporation, Milford, MA, USA) was used to determine the organic acid content. The temperature was 55 °C, the flow rate was 0.6 mL/min, and the mobile phase was 2.5 mmol/L H_2_SO_4_ [51]. Using colorimetry and the phenol–sodium hypochlorite technique, the ammonia nitrogen (NH_3_-N) concentration was determined [52]. The extract was gradually diluted, and the amounts of filamentous fungus, yeasts, coliform bacteria, and lactic acid bacteria were counted by cultivating them on rose Bengal medium, violet-red bile agar, and MRS agar, in that order [53].

### 4.4. Bacterial and Fungal Community Analysis

A cryogenic oscillator (THZ-98C, Shanghai Yiheng Scientific Instrument Co., Ltd., Shanghai, China) was used to shake the fresh samples (10 g) in 90 mL of sterile distilled water for 30 min at 4 °C and 180 rpm. The samples were then filtered through three layers of sterile gauze. To enrich the sediment, the filtrate was centrifuged for 15 min at 4 °C at 13,000× *g* in a cryogenic centrifuge (ST 16R, Thermo Fisher Scientific, Inc., Waltham, MA, USA). High-throughput sequencing was carried out on the sediments [54].

The FastDNA^®^ Spin Kit (MP Biomedicals, Solon, OH, USA) was utilized to extract total DNA from the bacteria and fungus present in the silage samples. Full-length fungal internal transcribed spacers (ITS) were amplified with specific forward ITS1F (CTTGGTCATTTAGAGGAAGTAA) and reverse ITS2R (GCTGCGTTCTTCATCGATGC) primers, while the hypervariable region V3–V4 of full-length bacterial 16S rRNA genes was amplified using PCR, with specific forward 341F (CCTAYGGGRBGCASCAG) and reverse 806R (GGACTACNNGGGTATCTAAT) primers [23]. The PCR products were purified using the AxyPrep DNA Gel Extraction Kit (Axygen Biosciences, Union City, CA, USA); the MiSeq PE300 platform of the Illumina Company was used for sequencing (Shanghai Majorbio Bio-Pharm Technology Co., Ltd., Shanghai, China), with a QuantusTM Fluorometer (Promega, Madison, WI, USA) being used for quantitative determination. Subsequently, raw sequences were spliced using FLASH (FLASH 1.2.11), and quality checking was handled via fastp. Moreover, sequences with 97% similarity were clustered in operational taxonomic units (OTUs) using UPARSE software (UPARSE 11). The microbiological diversity in each sample was evaluated using alpha diversity indexes, including the Chao richness estimator, Simpson diversity index, and Shannon diversity index. In order to evaluate the structural variation of the microbiota, beta diversity was examined. Principal coordinates analysis (PCoA) was then carried out [55]. The majorbio cloud platform, available online for free, was used to examine the data. The NCBI Sequence Read Archive database received the sequencing data (accession: PRJNA1000435).

### 4.5. Statistical Analysis

Using the General Line Model of SPSS (SPSS 22.0 program, SPSS Inc., Chicago, IL, USA), a factorial analysis of variance was used to examine the effects of the additives, the duration of aerobic exposure, and their interactions on the fermentation quality, microbial counts, and bacterial and fungal community indices of silage. Tukey multiple range tests were used to examine significant differences, with *p* < 0.05 indicating statistical significance.

## 5. Conclusions

Inoculation with *Lactobacillus buchneri* or *Bacillus licheniformis* maintained a good fermentation quality for whole-plant corn silage during the aerobic exposure phase, reduced the relative abundance of undesirable microorganisms, and improved aerobic stability. When silage was exposed to an aerobic environment, the fermentation products were not correlated with the bacterial community, but were significantly correlated with the fungal community. *Acetobacter*, *Paenibacillus*, and *Bacillus* participated in aerobic spoilage bacterial processes. *Issatchenkia*, *Monascus*, and *Tetrapisispora* were the dominant fungal communities in the late aerobic exposure period. Inoculation with *Lactobacillus buchneri* or *Bacillus licheniformis* slows down the succession of the fungal community from *Issatchenkia* to *Monascus*, thus delaying the aerobic spoilage process.

## Figures and Tables

**Table 1:** Chemical and microbial characteristics of fresh whole-plant corn.

Item	Whole-Plant Corn	SEM
DM (g/kg FW)	372.54	4.286
CP (g/kg DM)	79.84	0.541
aNDF (g/kg DM)	375.72	18.067
ADF (g/kg DM)	173.84	10.825
WSC (g/kg DM)	113.70	10.085
Ash (g/kg DM)	41.51	3.850
Lactic acid bacteria (lg CFU/g)	5.64	0.106
Coliform bacteria (lg CFU/g)	5.90	0.174
Yeasts (lg CFU/g)	6.39	0.019
Filamentous fungi (lg CFU/g)	<2.00	-

DM represents dry matter; FW represents fresh weight; CP represents crude protein; aNDF represents neutral detergent fiber assayed with a heat-stable amylase; ADF represents acid detergent fiber; WSC represents water soluble carbohydrates; SEM represents standard error of the mean.

**Table 2:** Nutrient composition of whole-plant corn silage after 60 days of ensiling.

Item	Treatments			SEM	*p* Value
	CK	LB	BL		
DM (g/kg FW)	371.05	374.90	374.33	3.173	0.869
CP (g/kg DM)	83.65	85.98	82.65	0.613	0.904
aNDF (g/kg DM)	352.37	343.40	340.00	11.480	0.766
ADF (g/kg DM)	171.33	171.60	169.87	6.520	0.993
WSC (g/kg DM)	46.93	39.83	39.25	1.382	0.089
Ash (g/kg DM)	46.53 a	41.60 ab	40.90 b	0.725	<0.05

Means with different lowercase letters in the same row (a,b) differ significantly (*p* < 0.05). FW represents fresh weight; DM represents dry matter; CP represents crude protein; aNDF represents neutral detergent fiber assayed with a heat-stable amylase; ADF represents acid detergent fiber; WSC represents water soluble carbohydrates; CK represents silage without inoculation; LB represents silage with inoculation of *Lactobacillus buchneri*; BL represents silage with inoculation of *Bacillus licheniformis*; SEM represents standard error of the mean.

**Table 3:** Variations in the richness and diversity of the bacterial community in whole-plant corn silage after aerobic exposure.

Item	D	Treatments			SEM	*p* Value		
		CK	LB	BL		D	T	D*T
Shannon	0	2.93 Aa	2.67 Ba	3.04 Aa	0.034	<0.01	<0.01	<0.01
	3	2.86 Aa	0.81 Bb	0.50 Bb				
	7	0.60 Ab	0.59 Ab	0.35 Bb				
Simpson	0	0.10 Bb	0.14 Ab	0.10 Bb	0.019	<0.01	<0.01	<0.01
	3	0.10 Bb	0.70 Aa	0.77 Aa				
	7	0.63 a	0.70 a	0.60 a				
Chao	0	281.53 Ba	387.13 Aa	350.13 Aa	3.933	<0.01	<0.01	<0.01
	3	217.60 Ab	164.03 Ab	59.20 Bb				
	7	10.92 Ac	8.25 Bc	8.63 Bb				
Coverage (%)	0	99.83 b	99.78 c	99.82 b	0.000	<0.01	0.464	0.130
	3	99.88 b	99.91 b	99.93 a				
	7	99.99 a	99.99 a	99.99 a				

Means with different uppercase letters in the same row (A,B) differ significantly (*p* < 0.05). Means with different lowercase letters in the same column (a–c) differ significantly (*p* < 0.05). CK represents silage without inoculation; LB represents silage with inoculation of *Lactobacillus buchneri*; BL represents silage with inoculation of *Bacillus licheniformis*. SEM represents standard error of the mean. D represents aerobic exposure days; T represents treatments; D*T represents interaction between treatments and aerobic exposure days.

**Table 4:** Variations in the richness and diversity of the fungal community in whole-plant corn silage after aerobic exposure.

Item	D	Treatments			SEM	*p* Value		
		CK	LB	BL		D	T	D*T
Shannon	0	2.68 a	2.94 a	2.89 a	0.037	<0.01	<0.01	<0.01
	3	2.91 a	2.57 b	2.96 a				
	7	0.99 Bb	1.53 ABc	2.09 Ab				
Simpson	0	0.21 Ab	0.14 Cc	0.18 Bab	0.009	<0.01	<0.01	<0.01
	3	0.11 Bb	0.20 Ab	0.13 Bb				
	7	0.68 Aa	0.41 Ba	0.24 Ba				
Chao	0	630.02 Ba	750.19 Aa	653.06 Ba	7.387	<0.01	<0.05	<0.01
	3	745.05 a	647.32 b	684.13 a				
	7	268.08 Ab	88.27 Bc	302.61 Ab				
Coverage (%)	0	99.66 b	99.62 b	99.71 b	0.000	<0.05	0.875	0.451
	3	99.66 b	99.67 b	99.66 b				
	7	99.86 a	99.91 a	99.86 a				

Means with different uppercase letters in the same row (A–C) differ significantly (*p* < 0.05). Means with different lowercase letters in the same column (a–c) differ significantly (*p* < 0.05). CK represents silage without inoculation; LB represents silage with inoculation of *Lactobacillus buchneri*; BL represents silage with inoculation of *Bacillus licheniformis*. SEM represents standard error of the mean. D represents aerobic exposure days; T represents treatments; D*T represents interaction between treatments and aerobic exposure days.

## Data Availability

The original contributions presented in the study are included in the article; further inquiries can be directed to the corresponding author.
